# Human Hunting and Nascent Animal Management at Middle Pre-Pottery Neolithic Yiftah'el, Israel

**DOI:** 10.1371/journal.pone.0156964

**Published:** 2016-07-06

**Authors:** Lidar Sapir-Hen, Tamar Dayan, Hamoudi Khalaily, Natalie D. Munro

**Affiliations:** 1 Department of Archaeology and Ancient Near Eastern Cultures, Tel Aviv University, Tel-Aviv, Israel; 2 The Steinhardt Museum of Natural History, Tel Aviv University, Tel-Aviv, Israel; 3 Department of Zoology, Tel Aviv University, Tel-Aviv, Israel; 4 Israel Antiquities Authority, Jerusalem, Israel; 5 Department of Anthropology, University of Connecticut, Storrs, Connecticut, United States of America; Chinese Academy of Sciences, CHINA

## Abstract

The current view for the southern Levant is that wild game hunting was replaced by herd management over the course of the Pre-Pottery Neolithic B period, but there is significant debate over the timing, scale and origin of this transition. To date, most relevant studies focus either on wild game exploitation in the periods prior to domestication or on classic markers of domestication of domestic progenitor species over the course of the PPNB. We studied the faunal remains from the 2007–2008 excavations of the Middle PPNB (MPPNB) site of Yiftah’el, Northern Israel. Our analysis included a close examination of the timing and impact of the trade-off between wild game and domestic progenitor taxa that reflects the very beginning of this critical transition in the Mediterranean zone of the southern Levant. Our results reveal a direct trade-off between the intensive hunting of wild ungulates that had been staples for millennia, and domestic progenitor taxa. We suggest that the changes in wild animal use are linked to a region-wide shift in the relationship between humans and domestic progenitor species including goat, pig and cattle.

## Introduction

There is growing consensus that the process of plant and animal domestication in southwest Asia was a multiregional phenomenon—that is, similar large-scale co-evolutionary processes occurred across a broad area, but played out differently at the local scale [1–6]. Investigating the co-evolution of human-animal relationships in the archaeological record, especially the initial stages, has proven to be a complex task [2, 7]. The current view for the southern Levant is that wild game hunting was replaced by herd management over the course of the Pre-Pottery Neolithic B period (PPNB: 10500–8250 cal, BP) [8–11], but there is significant debate over the timing, scale and origin of this transition. To date, most relevant studies focus either on wild game exploitation in the periods prior to domestication (e.g., [12–14]) or on classic markers of domestication such as taxonomic frequencies, mortality profiles and body-size of domestic progenitor species over the course of the PPNB [3, 9, 15–18]. We consider these classic markers in our presentation of new faunal data from the most recent excavations (2007–2008) of the Middle PPNB (MPPNB) site of Yiftah’el (8200–7700 cal BC) [19], but the primary contribution of this paper is its close examination of the timing and impact of the trade-off between wild game and domestic progenitor taxa (goat, pig and cattle) that reflects the very beginning of this critical transition in the Mediterranean zone of the southern Levant.

We examine the important moment when domestic progenitor species first begin to increase in south Levantine assemblages through the careful study of both the wild and domestic progenitor taxa in the diet. The scale of the impact of these domestic progenitors on human economies is measured by examining wild resource exploitation. In particular, we investigate changes in the intensity of human hunting by examining the relative abundance of high- and low-ranked wild taxa, and the mortality profiles and degree of bone fragmentation of the most common wild species, the gazelle. The timing, scale and “domestic status” of the domestic progenitors are tracked through their relative abundance, average body-size and mortality profiles. We set the results from Yiftah’el into their temporal context through comparison with new and published results from the EPPNB and MPPNB assemblages from Motza (Judean Hills, Israel) [13, 20], the only comprehensively studied EPPNB assemblage in the region. Although Motza is located about 100 km south of Yiftah’el, both sites are situated in Mediterranean settings close to alluvial deposits with good potential for early cultivation [21].

### Current Synopsis of Animal Exploitation in the Southern Levant

Intensive exploitation of hunted resources is well established in the Late Epipaleolithic (Natufian) periods of the southern Levant (ca. 15,000–11,700y BP) [12, 22–27]. In particular, it is marked by increased proportions of lower-ranked smaller-bodied ungulates, such as gazelles, in comparison to larger-bodied ungulate prey. Likewise, intensified use of mountain gazelle (*Gazella gazella*), the most hunted wild game taxon, is expressed by elevated frequencies of juvenile animals [12, 24, 28] and intensive carcass use [29]. Intensification is also evidenced by resource diversification, expressed by the addition of a variety of low-ranked small game taxa into human diets [12, 24, 26, 28, 30], especially high-cost taxa such as partridge and hare, at the expense of easy to capture species like tortoises [12, 22, 24, 25, 31]. Intensified resource use in the Epipaleolithic has been linked to human population packing, technological change and growing site occupation intensity (sedentism) [12, 24, 28, 30, 31] and is supported by numerous lines of independent archaeological evidence [32–35].

Detection of the early stages of the domestication process in the archaeological record is notoriously difficult because morphological changes are not expected until humans began to practice selective breeding. Instead, evidence for a changing relationship between people and domestic progenitor species is usually sought in increased frequencies of these taxa in the archaeological record [30, 36–38] and the selective culling of juvenile males reflected in herd mortality and sex profiles [39, 40]. In the Levant, the current consensus is that sheep (*Ovis aries*) first appeared in the northern Levant in the MPPNB but only expanded into the southern Levant in the LPPNB [3, 10, 11, 41–44], suggesting a delayed adoption pattern [45]. The picture is murkier for goats (*Capra hircus*). Some argue that goat followed a similar trajectory to sheep, but entered the region from the north earlier in the MPPNB [10, 42]. Others [9, 37, 46, 47] call upon gradual increases in the abundance of goat as evidence of local domestication starting in the MPPNB. Regardless, morphological changes in caprine populations are not detected until the LPPNB ([9], but see [47]). LPPNB increases in caprine exploitation are accompanied by an increase in the frequencies of other domestic progenitors, such as wild boar (*Sus scrofa*) and aurochs (*Bos primigenius*), but body-size and mortality data suggest that these taxa were not fully domesticated until the Pottery Neolithic (PN) [8]. Despite inter-site variation in the southern Levant, the proportion of domesticated animals dramatically increases only after the Late PPNB [37, 45], after which point they provide the economic basis of south Levantine diets.

Although the abundance of domestic progenitors species rises across the PPN, wild game still constitute a substantial portion of the economy. The study of resource diversity and exploitation intensity tracks the subtleties of Neolithic subsistence change that accompanied the domestication of plants and animals. At this turning point in human history, we trace changes in human behavior in relation to wild resource exploitation along a continuum of intensifying human-animal relations.

### The MPPNB site of Yiftah’el

**Fig 1:**
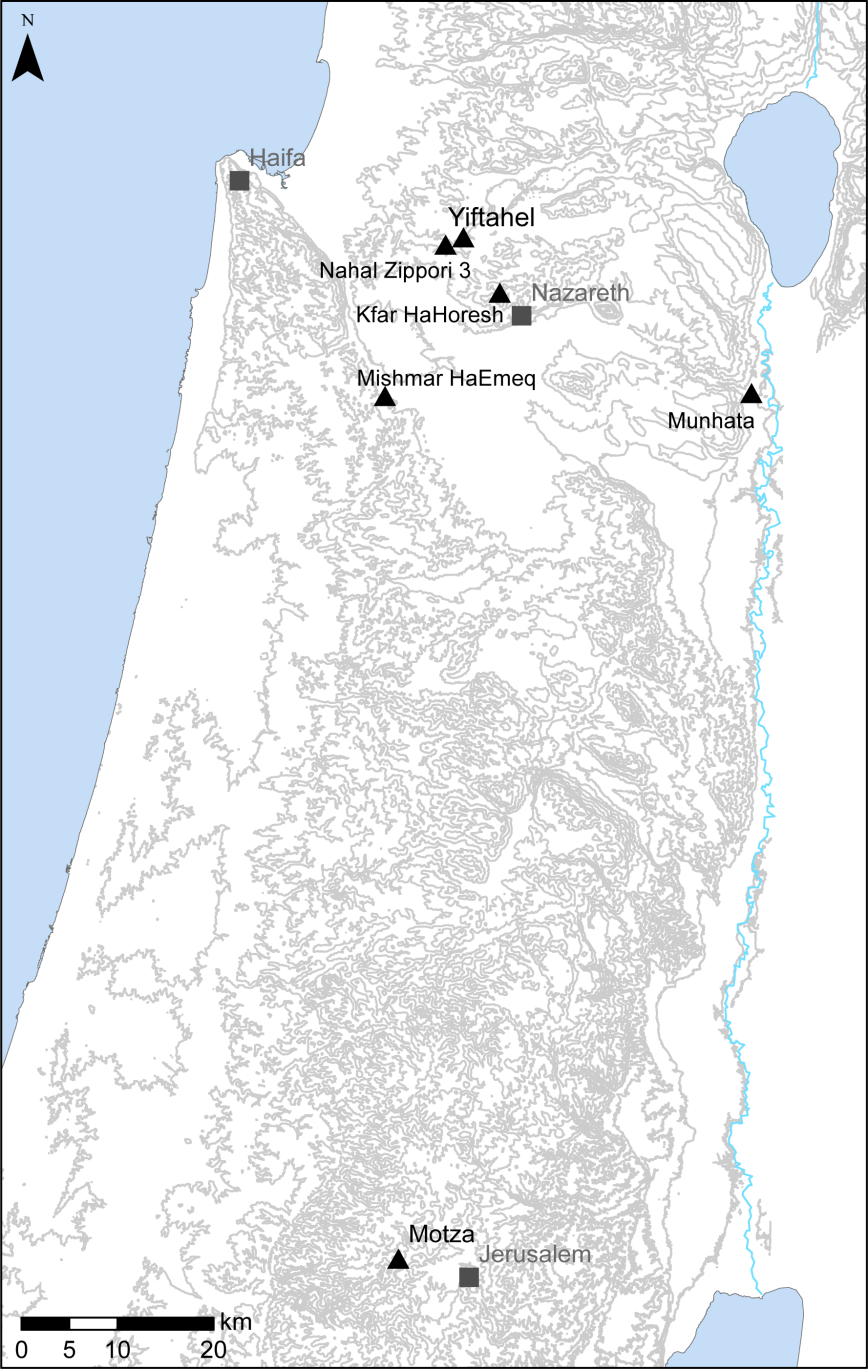
Location of Yiftah’el and Motza.

The 2007–2008 excavations at Yiftah’el were initiated in response to further expansion of Highway 77. The main occupational period represented in these excavations dates to the Middle PPNB (8,000–7,000 BC) [48]. Pottery Neolithic (Lodian and Wadi Rabah) and Bronze Age deposits were also uncovered but these are not included here. Four areas (F, G, H and I; Fig 1) were opened during the 2007–8 excavations—but this study focuses only on the MPPNB excavations in Areas G and I. The PPNB buildings are rectilinear in plan with interior features including constructed hearths, pits, installations, and burials. All have thick lime-plastered floors and mud-brick and/or stone walls. The material remains recovered from Areas G and I are extremely rich and include lithics, botanical and faunal remains among others [19, 49]. Notably, a cache of three plastered skulls deposited in a pit were recovered from Area I [50].

Horwitz [9] examined a contemporaneous MPPNB faunal assemblage from Areas C and D from Yiftah’el, as well as PPNC and Early Bronze Age assemblages from Areas A and B. These were excavated in 1982–3 by various expeditions (University of Haifa In 1982, IAA and Hebrew University in Jerusalem in 1983). Horwitz [9] examined the relative taxonomic abundance of key animal taxa in combination with mortality profiles and average body-size indices for *Gazella*, *Capra*, *Bos* and *Sus*. She argued that gradual increases in the exploitation of *Capra* reflected the initial stages of goat herd management in the MPPNB. After comparing her results with data from other MPPNB assemblages in the region, she concluded that this gradual increase provided better evidence for the autochthonous domestication of goats, than the introduction of managed herds from the northern Levant. Here, we delve more deeply into this question, by expanding sample sizes of domestic progenitor species and adding new lines of independent zooarchaeological evidence to determine the extent of the impact of these taxa on the wild diet.

The MPPNB from Yiftah’el was compared with new and published results from the EPPNB and MPPNB assemblages recovered from recent excavations at Motza near Jerusalem. These excavations revealed a large Neolithic site that was continuously inhabited from the EPPNB until the Pottery Neolithic period. The rare, well-dated EPPNB phase includes human burials, clay and stone figurines, and rich flint, obsidian and faunal assemblages [51]. Architectural remains from the MPPNB phase include small and narrow rectangular buildings with thick layers of repeatedly plastered floors.

## Methods

Material from both the MPPNB and Pottery Neolithic was recovered during the 2007–2008 excavations but only material from clean MPPNB contexts is included here. Bones were retrieved by hand, except for floor contexts which were sieved through 5mm mesh. All animal remains recovered from Levels G3 and I5 in Areas G and I from the 2007–2008 excavations at Yiftah’el were examined. Skeletal elements including cranial fragments, vertebrae, long bone articular ends and long bone shafts were identified to the closest possible taxonomic unit using the comparative collection stored at the Steinhardt Museum of Natural History of Tel Aviv University. Skeletal elements were divided into five zones (proximal and distal epiphysis, proximal and distal shaft, and mid-shaft) and the completeness of each zone was recorded for every specimen. The number of identified specimens (NISP) was used for taxonomic abundance analyses [52]. The minimum number of elements (MNE) was calculated by dividing the sum of the completeness scores for each zone by 100%—the best represented zone for each element represents its MNE [53]. MNE measures were used to calculate mortality profiles.

The intensity of fragmentation of the gazelle assemblage was recorded from a sample of gazelle long bone elements (n = 290) to investigate carcass exploitation intensity. The sample included all gazelle cortical specimens that encased long bone marrow. The morphology of the fracture angle, outline and edge were assessed, as well as the completeness of the shaft circumference following Villa and Mahieu [54]. Villa and Mahieu’s [54] study of human skeletons fragmented by known processes (food extraction versus post-depositional processes) identifies bone fracture characteristics that typify fresh versus old breaks. Fresh breaks suggest that bones were broken shortly after animals were killed, while dry breaks occur after collagen has decayed, often in response to post-depositional processes. Comparative data were also collected from EPPNB and MPPNB gazelle assemblages from Motza. Fragmentation intensity was assessed by investigating the %completeness (based on MNE) of bones with small but variable amounts of bone marrow (following [29]).

Gazelle mortality profiles were constructed using both long bone fusion and tooth wear data derived from the lower deciduous fourth premolar (dp_4_) and lower third molar (M_3_) that in combination cover the gazelle’s complete lifespan [55]. *Capra* mortality profiles were constructed following Zeder’s [56] long bone fusion stages and Payne’s [57] stages for tooth wear and eruption. Mortality profiles for *Sus* and *Bos* were based only on long bone fusion stages following Silver [58].

Measurements of complete long bone epiphyses of *Capra*, *Bos*, *Sus* and *Gazella* long bones were taken following von den Driesch [59]. Diachronic comparisons of *Capra*, *Bos* and *Sus* with other sites were carried out using the Log-Scale Index (LSI) [60]. Only one measurement per specimen was included in the LSI to avoid double counting, breadth measurements were chosen over depth measurements for consistency. Standard animal values for the LSI derive from published sources for wild goat [61], wild cattle [62] and wild pig [63]. Samples of *Gazella* distal humeri and the glenoid fossa of the scapula were large enough to directly compare gazelle body size between EPPNB Motza [20], and MPPNB Yiftah’el to track the trajectory of body size change over time. The comparison of single measurements is a more robust technique than the LSI that must combine multiple measurements to obtain sufficient sample size for analysis. The gazelle body size comparisons serve as a “control” for those of the domestic progenitors to determine if body size change is related to human control or to environmental factors such as climate. If body size change in the domestic progenitors differs from wild taxa, the change is clearly determined by independent factors.

### Ethics Statement

The excavations, undertaken on behalf of the Israel Antiquities Authority (permit 5252) and underwritten by the Israel National Roads Company (Department of Public Works), was directed by one of the authors (H. Khalaily). The zooarchaeological specimens (catalogue numbers #1–6381) can be accessed at the Zooarchaeological Collections of the Steinhardt Museum of Natural History at Tel Aviv University.

## Results

### Relative taxonomic abundance

A total of 4230 (NISP) bone specimens from the Yiftah’el MPPNB assemblage were identified to taxon and element (Table 1). The assemblage is dominated by ungulates (86%), but includes a small proportion of small game (5%) and carnivores (9%). Gazelle is the most common taxon in the assemblage and dominates the ungulate fraction (69% of ungulates identified to species) (Table 2). In order of declining abundance, the remaining ungulates include pig (*Sus scrofa*; 14%), cattle (*Bos* sp.; 11%), goat (*Capra* sp.; 7%), and red deer (*Cervus elaphus*; <1%). The relative abundance of gazelle is similar to MPPNB Motza (69%), but notably lower than the EPPNB levels at Motza (83%) (Table 2). The small game fraction at Yiftah’el is composed of Mediterranean spur-thighed tortoise (*Testudo graeca*; 3%), cape hare (*Lepus capensis*; 1%), and a few chukar partridges (*Alectoris chukar*; <1%). The relative abundance of small game (6.3%) is notably lower than the EPPNB and MPPNB levels at Motza (Table 2). The carnivore assemblage is composed mainly of red fox (*Vulpes vulpes*; 7%; 85% of carnivores).

**Table 1:** Number of identified specimens (NISP) for all taxonomic groups identified at Yiftah'el.

	Taxon	NISP
Ungulates	*Gazella gazella*	1974
	*Sus scrofa*	383
	*Bos primigenius*	304
	*Capra aegagrus*	182
	Cervidae	18
	*Cervus elaphus*	1
	Body Size A	59
	Body Size B	607
	Body Size C	54
	Body Size B/C	35
	**Total**	**3617**
Carnivora	*Vulpes vulpes*	316
	*Felis* sp.	34
	*Martes foina*	10
	*Canis familiaris*	7
	*Herpestes ichneumon*	4
	*Meles meles*	2
	Mustelidae	1
	**Total**	**374**
Small game	*Testudo graeca*	137
	Aves General	4
	Passeriformes	15
	*Alectoris chukar*	29
	Falconiformes	1
	*Lepus capensis*	53
	**Total**	**239**
Total		4230

Body size A = *Bos*, *Cervus*, *Dama*, *Sus-sized*; B = *Capra sized*; C = *Gazella sized*.

**Table 2:** NISP totals for taxonomic groupings used in relative taxonomic abundance analyses.

	Motza EPPNB		Motza MPPNB		Yiftah'el MPPNB	
Taxonomic Group	NISP	%NISP	NISP	%NISP	NISP	%NISP
*Gazella*	2881	82.6	289	69.1	1974	69.0
Cervidae	5	0.1	0	0.0	19	0.7
*Sus*	358	10.3	60	14.4	383	13.4
*Bos*	100	2.9	18	4.3	304	10.6
*Capra*	144	4.1	51	12.2	182	6.4
Total Ungulate Species	3488		418		2862	
Tortoise	313	53.5	136	89.5	137	57.6
Birds	21	15.6	6	5.9	48	20.1
Hare	181	30.9	7	4.6	53	22.3
Total Small Game	515		149		238	
Wild Ungulates	2886	82.7	289	69.1	1993	69.6
Dom Progenitor Ungulates	602	17.3	129	30.9	869	30.4
Total Ungulates	3488		418		2862	
Ungulates	3488	79.0	418	72.0	2862	82.4
Carnivores	742	11.6	82	9.7	374	10.8
Small Game	515	9.4	149	18.3	238	6.9
Total NISP	4745		649		3474	

### Wild game

**Fig 2:**
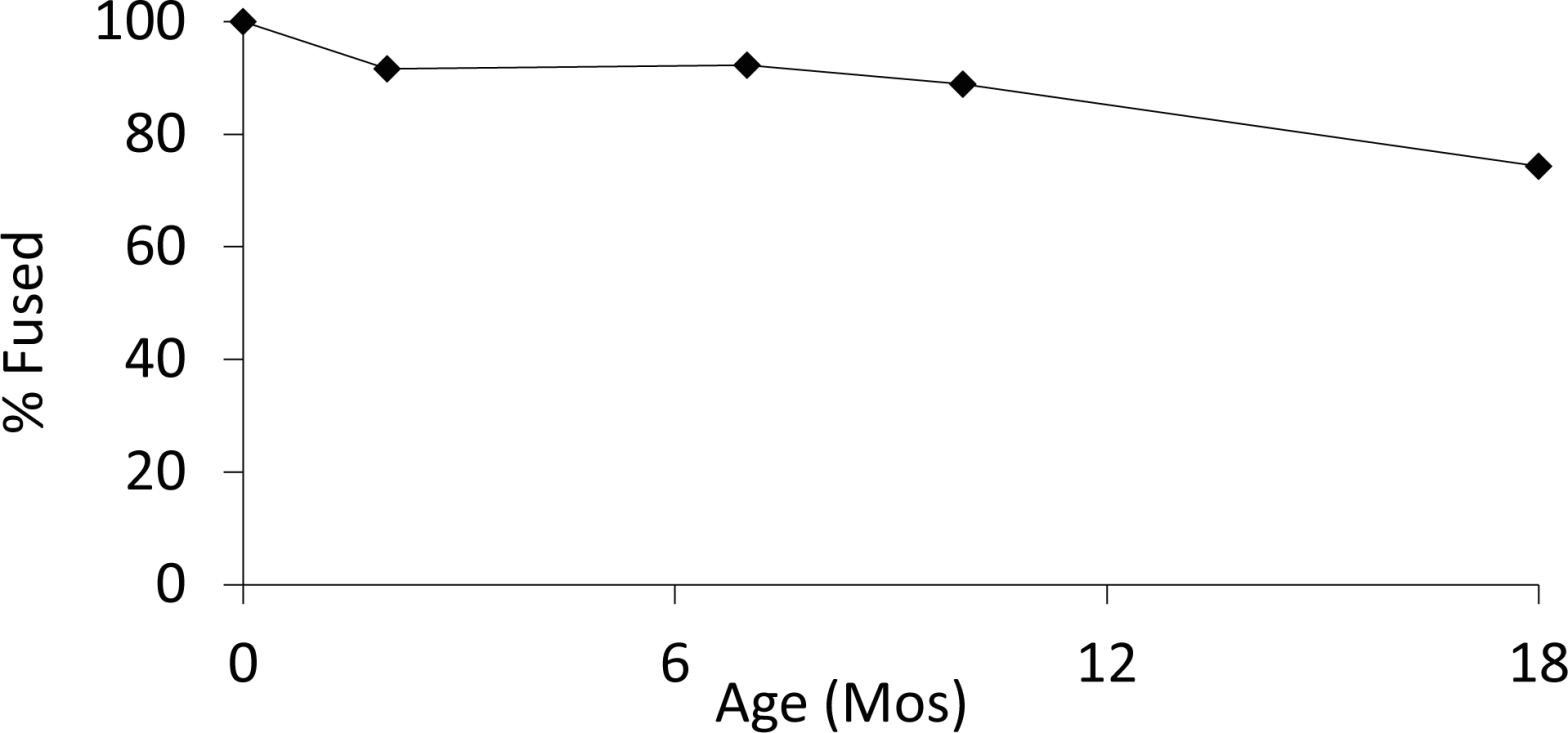
Gazelle survivorship based on fusion data (n = 672). Age axis indicates age at which elements included in sample for that point fuse (following [55]).

**Fig 3:**
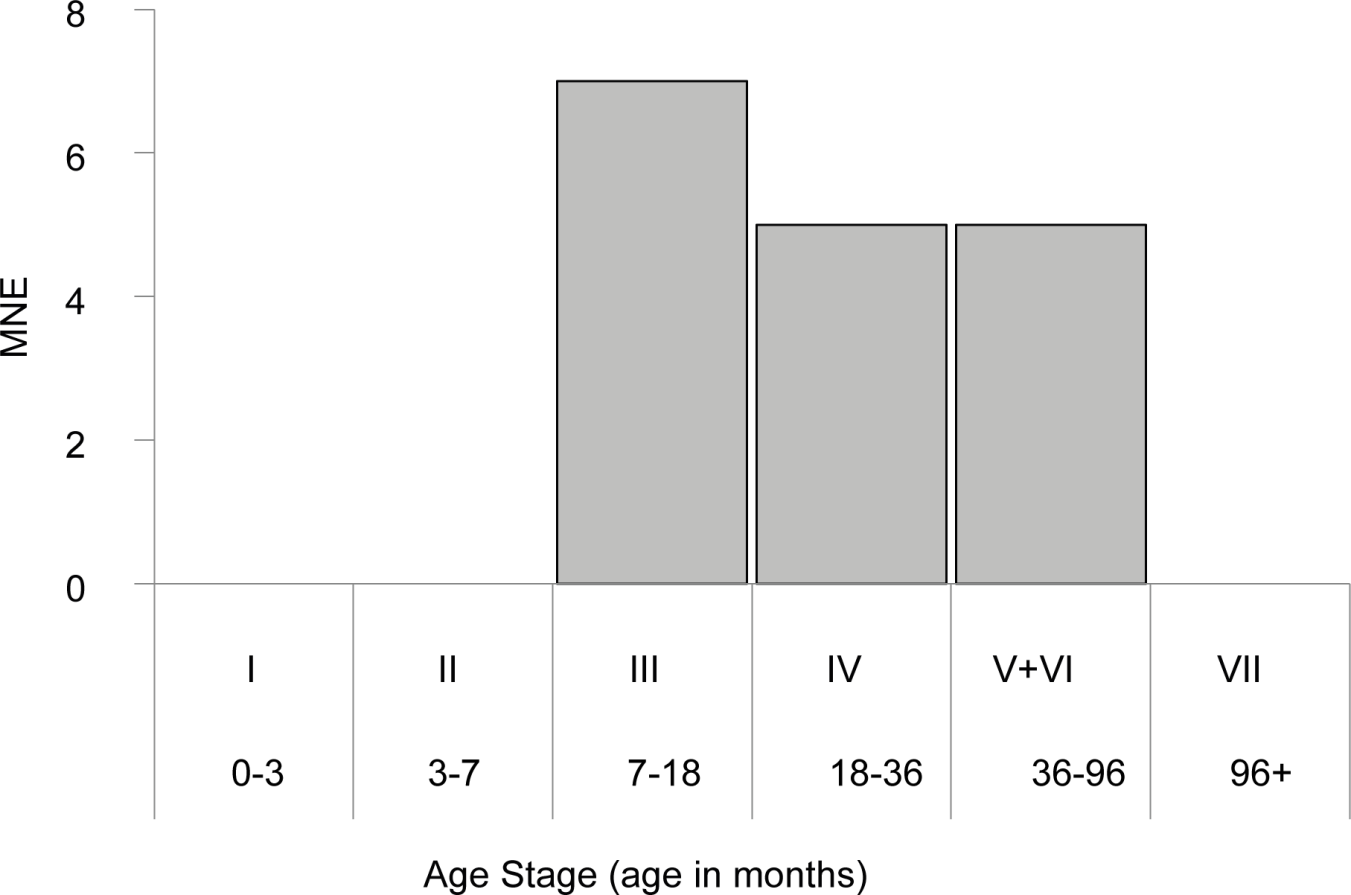
Gazelle mortality profile (n = 17) based on Munro et al.'s ([55]) tooth wear and eruption stages.

The gazelle long bone fusion data indicate a gradual decrease in survivorship up until the age when gazelles reach adulthood (18 months). Only 26% of gazelles were culled by this point (Fig 2; Table A in S1 Table for age data). This frequency is similar to that observed at EPPNB Motza [13]. The gazelle tooth wear analysis reveals similar proportions of juveniles, but the tooth sample is too small to be conclusive (n = 17; Fig 3; Table B in S1 Table). In all cases these mortality curves indicate a clear prime-aged bias—in stable gazelle populations about 33% of the animals are juveniles [64]. This pattern differs significantly from Natufian assemblages that are routinely juvenile dominated (>40% gazelles younger than 18 months at Hayonim Cave and Terrace, based on the fusion of the distal metapodial [12, 65]).

High percentages of oblique fracture angles (53%), curved outlines (66%) and smooth edges (78%) (see [54] for definition and illustrations of fracture types) indicate that the majority of fragmented gazelle long bones from Yiftah’el were broken when fresh (Table 3). In addition, high proportions of complete long bone circumferences (64%) (following [54]) indicate that many long bone shafts were never opened to access marrow. A re-examination of the gazelle assemblage from EPPNB Motza shows a similar result (62% complete) (Table 3). This value is significantly higher than those recorded in south Levantine Epipaleolithic assemblages (11–25%) ([29]: Table 2).

**Table 3:** Fracture angle, outline, edge and circumference values of gazelle long bone shaft fragments from MPPNB Yiftah'el.

**Angle**	**oblique**	**right**	**oblique and right**	**TOTAL**
MPPNB Yiftah'el	53 (153)	17 (49)	30 (88)	290
**Outline**	**spiral (curved)**	**transverse**	**intermediate**	**TOTAL**
MPPNB Yiftah'el	66 (192)	15 (43)	19 (55)	290
**Edge**	**smooth**	**jagged**	**intermediate**	**TOTAL**
MPPNB Yiftah'el	78 (222)	22 (61)	0 (1)	284
**Circumference**	**<1/2**	**>1/2**	**comp**	**TOTAL**
MPPNB Yiftah'el	18(53)	18(50)	64 (185)	288
EPPNB Motza	16(30)	22(42)	62(120)	192

Comparative shaft circumference data from EPPNB Motza. Values outside parentheses are = percentages, inside parentheses are = sample size. Fracture terms and definitions follow Vila and Mahieu [54].

Finally, gazelle foot bones have similar %completeness values despite containing varying quantities of bone marrow (Table 4). Astragali which contain no marrow are 93% complete (MNE = 52) while first (MNE = 143) and second (MNE = 84) phalanges are 87 and 95% complete respectively despite the presence of small quantities of bone marrow. These results suggest that humans were willing to invest only limited additional effort to extract small amounts of marrow in contrast to peoples of the Epipaleolithic periods [66]. Similarly, no correlation between fragmentation (MNE/NISP) and gazelle's marrow index [66] was found at EPPNB Motza [13].

**Table 4:** MNE, element completeness and marrow index for gazelle foot bones with small but varying amounts of bone marrow. Bones are considered complete if more than 90% of the bone is present. Marrow index values from Bar-Oz and Munro [66].

Element	MNE	>90% complete	Marrow Index
1st phalanx	143	87.41	3.02
2nd phalanx	84	95.23	0.55
3rd phalanx	60	83.33	0
Astragalus	52	92.3	0
Calcaneus	56	64.28	0

Two measurements have sufficient sample sizes to investigate the change in gazelle body-size between EPPNB Motza and MPPNB Yiftah’el—the distal breadth (Bd) of the humerus (Motza n = 47, YIFT n = 47) and the breadth (BG) of the glenoid fossa of the scapula (Motza n = 40; Yift n = 38). The results show that the Yiftah’el gazelles are similar in size (scapula: t = -1.47, p = 0.14) or larger (distal humerus: t = -2.87, p<0.001) than the gazelles from EPPNB Motza (Table A in S2 Table for raw measurements; Fig A in S1 Fig).

### Domestic Progenitor Species

#### Capra

**Fig 4:**
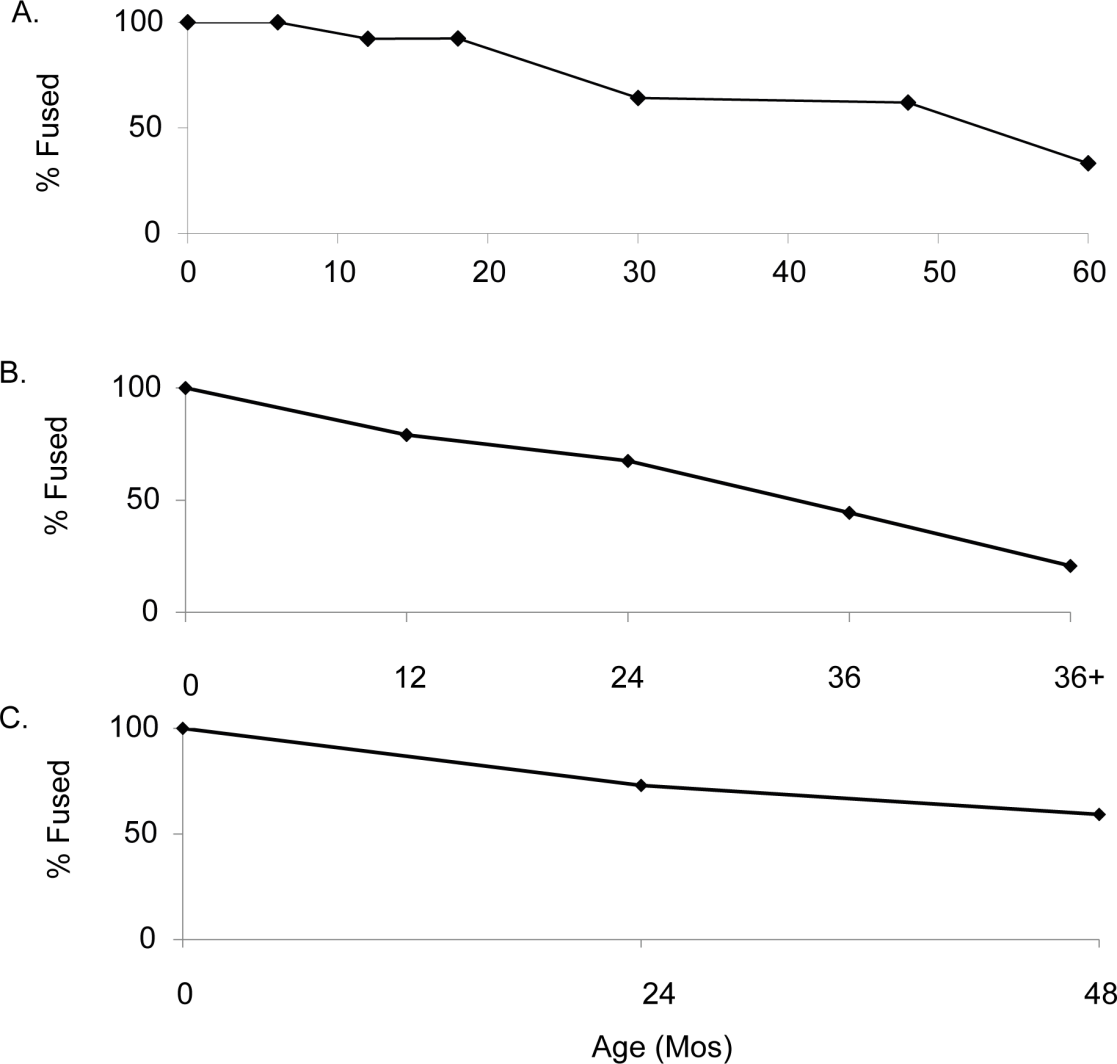
**Survivorship of (A) *Capra* (n = 317) [56]. (B) *Sus* (n = 127) [58]. (C) *Bos* (n = 90) [58].** Based on fusion data. Y-axis indicates proportion of fused specimens for elements that fuse by that age.

**Fig 5:**
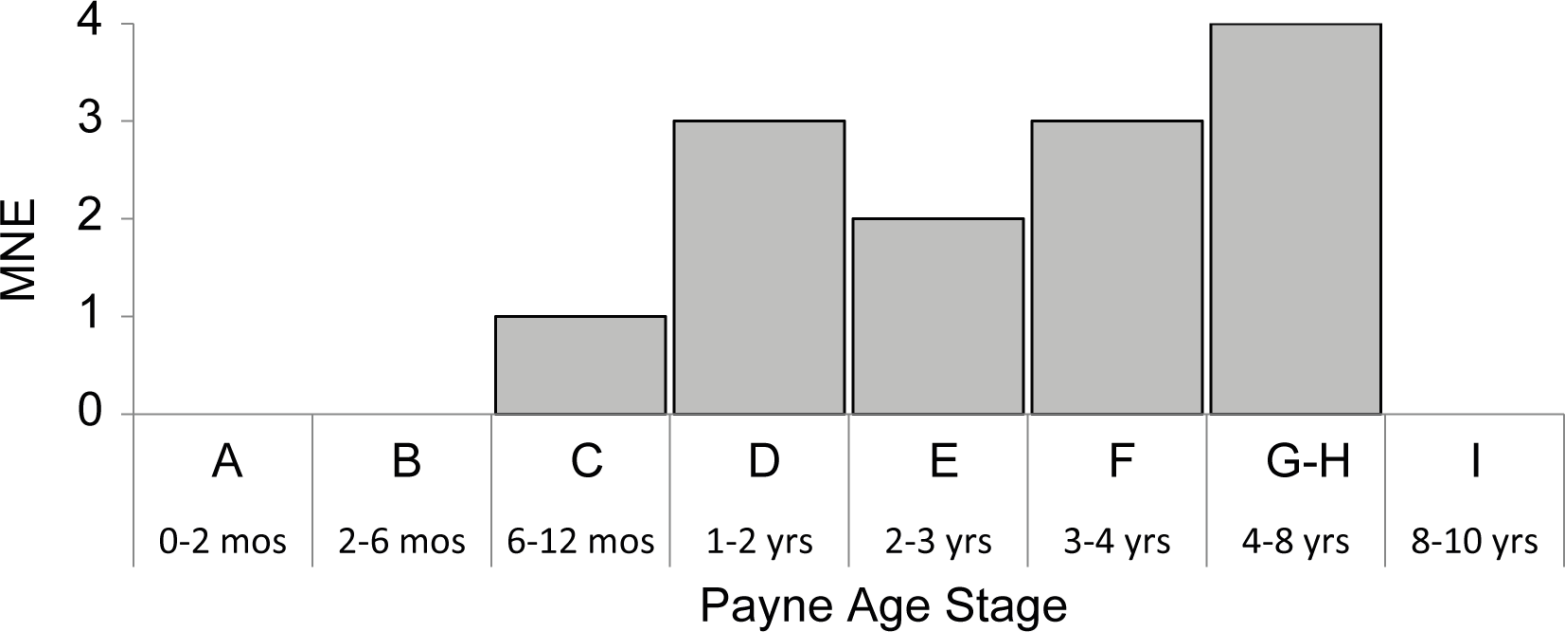
*Capra* mortality profile (n = 13), based on Payne's [57] tooth wear and eruption stages.

The morphology of five goat horn cores that could be identified to species is wild in form [47, 67]. The survivorship curve constructed from *Capra* long bone fusion data (Fig 4A; Table C in S1 Table) reveals that very few animals (8%) were slaughtered before 18 months of age. A dip in goat survivorship between the ages of 20 to 30 months is evident, but only 38% of the herd is culled before 36 months of age. Tooth wear analysis shows similar results, although the sample is small (Fig 5; Table D in S1 Table).

**Fig 6:**
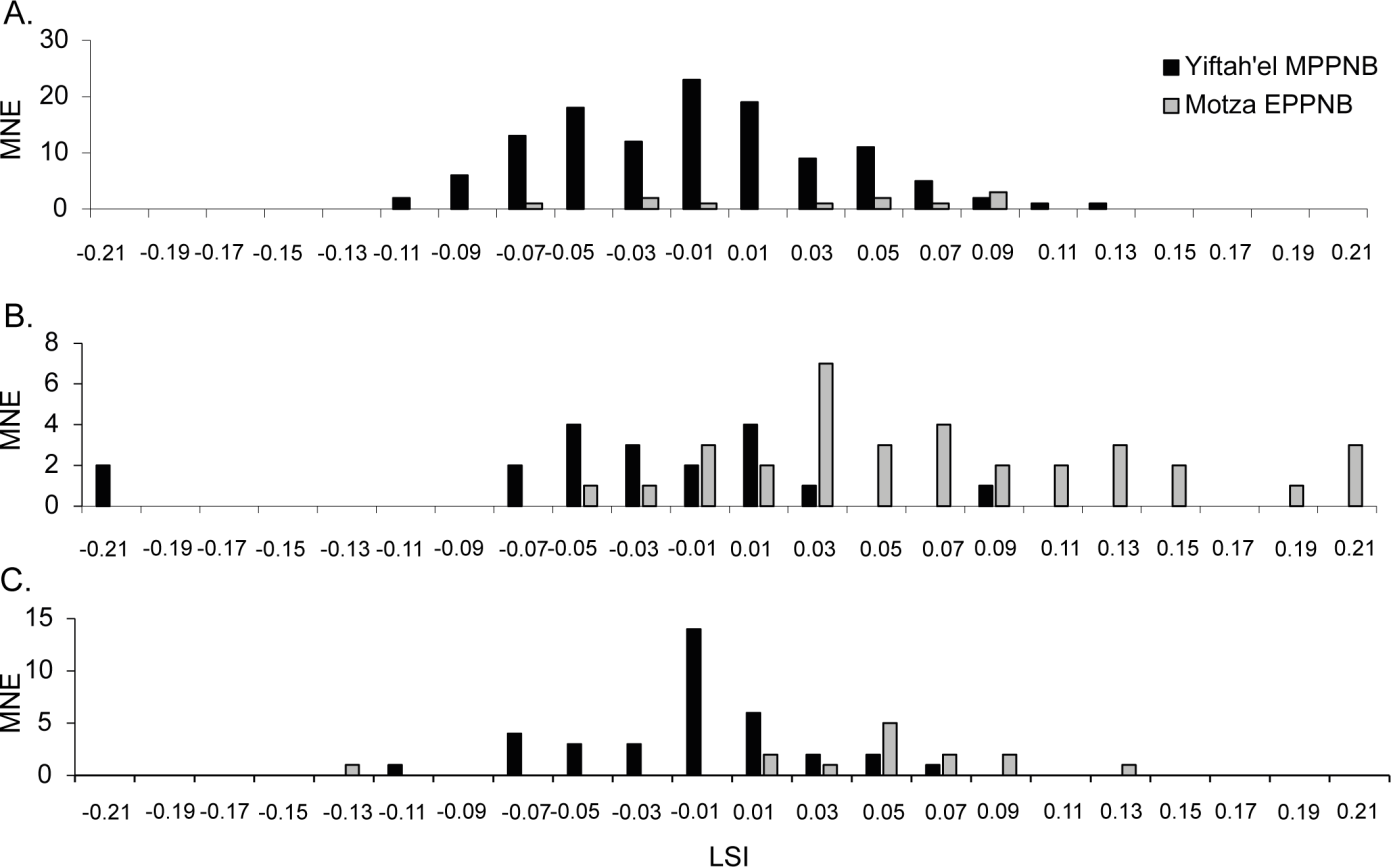
LSI distribution of (A) *Capra* (MPPNB Yiftah'el n = 122; EPPNB Motza n = 11). (B) *Sus* (MPPNB Yiftah'el n = 19; EPPNB Motza n = 34). (C) *Bos* (MPPNB Yiftah'el n = 36; EPPNB Motza n = 14).

The LSI profile (n = 122; Table B in S2 Table for raw measurements) for the Yiftah’el goats indicates a slight decrease in average body-size compared to published measurements from contemporary or earlier sites in the Mediterranean Hills including Natufian Eynan, EPPNB Motza and MPPNB Abu Ghosh and even a previous, but smaller sample from Yiftah’el (n = 13) (Table 5A; LSI range and mean as calculated in [3]). Where statistical analysis was possible, the size difference was significant (based on LSI, compared to EPPNB Motza, t = 3.08, p = 0.002) (Fig 6A). In any event, this reduction in body-size is minimal compared to that expressed in fully domesticated goats from for example, the site of Iron Age Tel Dor (t = 5.44, p<0.001; Table 5A). This decrease in size is accompanied by a more positive skewness in *Capra* measurements from Yiftah’el compared to EPPNB Motza (2nd Pearson's skewness coefficient for log-transformed measurements = -0.01 for Yiftah’el, -0.7 for Motza), indicating a shift in Yiftah’el assemblage toward smaller-bodied animals, likely females [68].

**Table 5:** Mean, upper and lower ranges (mean+/- 1 SD) of *Capra*, *Sus* and *Bos* LSI values for Yiftah'el, Motza and other applicable sites form the region. Size range and Mean, based on LSI values.

*Capra*	Site	Period	Lower Range	Mean	Upper Range	N
	Eynan*	Early Natufian	-0.0003	0.034	0.0683	32
	Motza	EPPNB	-0.023	0.0374	0.0983	11
	**Yiftah'el**	**MPPNB**	**-0.0599**	**-0.011**	**0.0379**	**122**
	Yiftah'el (Horwitz)*	MPPNB	-0.0399	0.019	0.0779	13
	Abu Ghosh*	MPPNB	-0.0229	0.021	0.0649	284
	Tel Dor**	Ir-Rm	-0.0642	-0.031	0.0013	69
*Sus*	Site	Period	Lr Range	Mean	Up Range	N
	Motza	EPPNB	0.0035	0.0717	0.1398	34
	**Yiftah'el**	**MPPNB**	**-0.1185**	**-0.00406**	**0.0372**	**19**
*Bos*	Site	Period	Lr Range	Mean	Up Range	N
	Motza	EPPNB	-0.0139	0.0445	0.103	14
	**Yiftah'el**	**MPPNB**	**-0.0508**	**-0.0093**	**0.0322**	**36**

Period abbreviations indicate Early Nat = Early Natufian, Ir-Rm = Iron-Roman.

* Data from Martin and Edwards [3] is not primary but summarized from original sources

**Data from Tel Dor is based on Sapir-Hen [69]. a. *Capra*. b. *Sus*. c. *Bos*

#### Suids

After gazelle, Suids are the most common animal at Yiftah’el. The survivorship curve based on bone fusion data (n = 127; Fig 4B; Table E in S1 Table) reveals that 75% of pigs survived to two years of age while only 45% made it to three years. This culling profile is similar to that reported for the wild pig populations from the early levels at Çayönü Tepesi but differs from populations in the later levels that are described as domestic [63]. There are also fewer juveniles than reported for PPNC Sha’ar Hagolan [70].

The Suid LSI values (n = 19; Table C in S2 Table for raw measurements) reveal a notable decline in both the average and range of bone measurements compared to the Suids from EPPNB Motza (t = 5.46, p<0.001; Table 5B, Fig 6B), but are similar in size to those reported from MPPNB Abu Ghosh (of the two reported measurements, only the Humerus Bd Measurement is comparable [15].

#### Bos

Survivorship curves based on *Bos* bone fusion data (n = 90) show that almost 80% of the herd survived until three years of age, but only 33% survived to four years of age (Fig 4C; Table F in S1 Table). The survivorship curve is similar to that reported for the Pottery Neolithic layers of Sha’ar Hagolan [70]. However, sample sizes in both assemblages are small.

LSI data of *Bos* measurements from Yiftah’el (n = 36; Table D in S2 Table) reveal a significant decrease in the range and average size of skeletal measurements compared to *Bos* from EPPNB Motza (t = 3.66, p<0.001; Table 5C, Fig 6C). The cattle are similar in size to *Bos* from MPPNB Abu Ghosh [15] and PPNC Sha’ar Hagolan [70].

## Discussion

Our study of the MPPNB faunal assemblage from Yiftah’el and comparisons with the EPPNB assemblage of Motza reveal a clear trade-off between intensive wild resource exploitation and domestic progenitor taxa. The scale of the impact of increased domestic progenitors on wild resource exploitation in association with small changes in the body size of the three domestic progenitor species suggest that the introduction of these species does not simply reflect new hunting practices, but represents a change in the relationship between humans and specific animal taxa.

The representation of domestic progenitor species increases from 17% at EPPNB Motza to 30% in the MPPNB of both Motza and Yiftah’el. This increase represents a significant rise in the frequency of each of the three domestic progenitor species-taxa, that each make only small contributions to Epipaleolithic assemblages. Importantly, introduction of new progenitor species coincides with a release in hunting pressure on wild resources at Yiftah’el in comparison to the preceding EPPNB assemblage from Motza. This is evident in the results of four independent measures of hunting intensity on wild game taxa: a) a reduction in the number of small bodied-gazelle in comparison to larger ungulates, namely domestic progenitor species; b) a decline in the abundance of small game in comparison to ungulates; c) a decline in the abundance of juvenile gazelles; and d) less intensive processing of phalanges for bone marrow. The latter two changes can already be observed in the EPPNB assemblage, and continue into the MPPNB. All of these indices reveal a shift from more to less costly hunting and extraction behaviors and thus an increase in foraging efficiency. This is indicated by the shift toward larger meat packages (from juvenile to adult gazelle; small to larger ungulate; small game to ungulates and small to large marrow stores) that provide higher returns for the energy invested. Together these results provide robust evidence for a gradual release in hunting pressure starting in the EPPNB [12, 28, 29].

The direct trade-off between the intensive hunting of wild ungulates that had been staples for millennia, and domestic progenitor taxa suggest that the changes in wild animal use are linked to a region-wide shift in the relationship between humans and domestic progenitor species including goat, pig and cattle. A gradual increase in the relative abundance of *Capra* from the MPPNB onward has long been noted in the southern Levant [9, 71, 72], although researchers debate the scale of its economic contribution, its geographic point of origin and whether or not it was managed during the MPPNB. Horwitz and colleagues [9, 15, 16] have been most vocal in their support of a local origin of goat management starting in the MPPNB. In the Mediterranean Hills region they support their argument with assemblages from Abu Ghosh, close to Motza, and a sample from previous excavations at Yiftah’el [9, 15]. The MPPNB assemblage from Abu Ghosh contains unusually high proportions of *Capra* (51% of ungulates), about half (46%) of which were culled before 36 months of age [15]. These goats are similar in size to PPNA counterparts, although they are slightly smaller than Epipaleolithic populations. Ducos and Horwitz [16] interpret this size reduction as a response to climate change. Goats comprise 16% of the ungulates in the sample from Garfinkel’s 1982–3 excavations from the MPPNB at Yiftah’el [9]. The proportion of juvenile animals (43% culled by 36 months) and their average body-size is similar to those from Abu Ghosh. Horwitz [15] concluded that the mortality profile does not indicate selective culling for meat typical of full domestication, but suggests an early attempt at cultural control of goats or selective hunting of wild prey. Our results from Yiftah’el are similar, but differ in the smaller size and slightly older age of the goat population—these small differences may relate to differences in sample sizes (Horwitz' measurements n = 13, age data n = 104; versus our measurements n = 122, age data n = 317).

Although juvenile animals are well represented at Abu Ghosh and both Horwitz’s and our sample from Yiftah’el (38–46% culled by 36 months), the proportion of juvenile individuals is still significantly lower than predicted by the classic models for a herd optimized for meat production (70% culled by 36 months of age; [57, 73]). In contrast, the Mediterranean Hills sites and our sample from Yiftah’el fit better with the mortality profile of a wild population of *Capra aegagrus* from Pakistan (a region with similar summer and winter temperatures to Israel, but lower precipitation) in which 40% of the population was culled prior to 36 months of age [74].

The trajectory of body size change for the wild gazelle (increase or steady) from the EPPNB of Motza to the MPPNB of Yiftah’el differs from that of two of the domestic progenitors–*Sus* and *Capra* (decrease) indicating that body-size change in these different taxa responded to independent processes. Body-size diminution is a typical indicator of herd animal domestication, but in the early stages of management it often derives from a shift in the adult sex ratio toward more females [40, 75]. This occurs because meat optimization requires that most males be slaughtered before they reach adulthood. Because unfused bones are often not measured, adult females are more likely to be reported in LSIs of herds managed for meat. This effect could not have been strongly pronounced at Yiftah’el since only 40% of the population was culled before three years of age, but it could have had a small effect as suggested by more positive skewing of the LSI compared to earlier assemblages, indicating more adult females in the population.

The increased prominence of *Capra*, and its small reduction in body-size suggest a change in the relationship between humans and goat herds that goes beyond a new hunting strategy at Yiftah’el. The difference in the trajectory of body size change in wild versus domestic progenitor taxa, further supports the observation that domestic progenitors are responding to different processes. Nevertheless, the survivorship curve for *Capra* does not reveal the scale of sex-specific juvenile culling typical of managed herds. The body-size data also indicate that some very large wild goats are present in the population. This pattern fits a general trend observed in other MPPNB sites [2, 3, 9], where a broad range of sizes and complex patterning are evident, indicative of local differences in hunting or potentially herd management strategies [9]. Thus, the goats do not provide evidence for classic herd management, but their increased frequency, associated reductions in hunting intensity and declines in average body-size suggest that humans had begun to exert control over these herds (see below).

Interestingly, *Sus* and *Bos* are more abundant than *Capra* in the Yiftah’el assemblage. Both taxa increase in abundance and reveal a small decline in average body-size in the MPPNB compared to the EPPNB at Motza. Their average body-size is more similar to that of cattle and pigs from the PPNC sites of Sha’ar Hagolan in the Jordan Valley. Nevertheless, neither of the age profiles show signs of selective culling. The age profile for *Sus* is similar to that of a hunted wild population, while the mortality data for *Bos* show a focus on older ages, typical of hunting or of herd management strategies in much later periods (such as the PN of Sha’ar Hagolan). More significant increases in the frequency of *Bos* and *Sus* have been noted for the PPNC [76, 77], and have been argued by Marom and Bar-Oz [76] to provide the earliest evidence for early herd management in the southern Levant. The trajectory toward domestication in the Mediterranean Hills may differ somewhat from the Jordan Valley. Although the case is not as clear as for caprines, we suggest that the *Sus* and *Bos* data from Yiftah-el also hint at a new relationship with ungulate taxa that were amenable to human control.

Overall, results from Yiftah’el, in particular the simultaneous increase in all three domestic progenitor species, the de-intensification of wild prey hunting and processing strategies that accompanies their entry into human diets and the small declines in the body-size of progenitor species that may indicate higher proportions of adult females in the population, support the emergence of a new relationship between humans and herd animals based on some kind of human control that preceded more obvious evidence for managed culling. Perhaps some females were confined by humans to improve their accessibility, decrease their cost of acquisition, and provide short-term storage on the hoof, but these animals were not efficiently culled. The gradual trajectory of this change that started in the EPPNB also suggest that this was a local development [9], supporting the growing perception that animal domestication was a multiregional phenomenon. However, this local process need not have been entirely independent, but likely involved an ongoing exchange of knowledge from the northern Levant [8, 9]. In the case of pig and cattle, these early steps did not quickly evolve into management, but instead reflect new interest in species that had appropriate qualities for human manipulation. This scenario does not rule out the possibility that new and potentially improved goat, pig or cattle herds were imported from the north and contributed to or replaced locally managed animals in later periods as was the case for domestic sheep [3].

## Supporting Information

S1 Fig Distribution of *Gazella* measurements from EPPNB Motza and MPPNB Yiftah’el. (DOCX)

S1 Table *Gazella*, *Capra*, *Sus*, *Bos* Age data from MPPNB Yiftah'el (DOCX)

S2 Table Raw measurements for *Gazella*, *Capra*, *Sus* and *Bos* from MPPNB Yiftah'el. (DOCX)
