# Supplementary material for: Human Hunting and Nascent Animal Management at Middle Pre-Pottery Neolithic Yiftah'el, Israel
Source: PLoS One. 2016 Jul 6;11(7):e0156964. doi: 10.1371/journal.pone.0156964 (PMC4934702; doi:10.1371/journal.pone.0156964)
Supplement: S1 Fig — (DOCX) [file pone.0156964.s001.docx]

**S1 Fig. Distribution of *Gazella* measurements from EPPNB Motza and MPPNB Yiftah’el.**

Figure A: Frequency distribution of breadth measurements of *Gazella* distal humeri (Bd) from EPPNB Motza and MPPNB Yiftah’el.

Figure B: Frequency distribution of breadth measurements of the glenoid fossa (BG) of *Gazella* scapulae from EPPNB Motza and MPPNB Yiftah’el.
