# Supplementary material for: Human Hunting and Nascent Animal Management at Middle Pre-Pottery Neolithic Yiftah'el, Israel
Source: PLoS One. 2016 Jul 6;11(7):e0156964. doi: 10.1371/journal.pone.0156964 (PMC4934702; doi:10.1371/journal.pone.0156964)
Supplement: S1 Table — (DOCX) [file pone.0156964.s002.docx]

**S1 Table. Age data from MPPNB Yiftah'el**

**Table A.** *Gazella* bone fusion data from MPPNB Yiftah’el.

|  | MNE | MNE | MNE |  | Age at |
| --- | --- | --- | --- | --- | --- |
| Bone Portion | Unfused | Fused | Total | %Fused | Fusion (mos) |
| Radius-Proximal | 2 | 22 | 24 | 91.7 | 0 to 7 |
| TOTAL Stage 1 | 2 | 22 | 24 | 91.7 | 0 to 7 |
| 1st Phalanx-Prox | 7 | 127 | 134 | 94.8 | 3 to 7 |
| 2nd Phalanx-Prox | 1 | 21 | 22 | 95.5 | 3 to 7 |
| Humerus-Distal | 1 | 68 | 69 | 98.6 | 3 to 7 |
| Pelvis-Acetabulum | 3 | 28 | 31 | 90.3 | 3 to 7 |
| Scapula-Glenoid | 12 | 45 | 57 | 78.9 | 3 to 7 |
| TOTAL Stage 2 | 24 | 289 | 313 | 92.3 | 3 to 7 |
| Tibia-Distal | 3 | 24 | 27 | 88.9 | 3 to 18 |
| TOTAL Stage 3 | 3 | 24 | 27 | 88.9 | 3 to 18 |
| Femur-Proximal | 4 | 19 | 23 | 82.6 | 7 to 18 |
| Calcaneum | 31 | 47 | 78 | 60.3 | 7 to 18 |
| Metapodial-Distal | 18 | 76 | 94 | 80.9 | 7 to 18 |
| Femur-Distal | 9 | 19 | 28 | 67.9 | 7 to 18 |
| Ulna-Proximal | 8 | 19 | 27 | 70.4 | 7 to 18 |
| Humerus-Proximal | 2 | 16 | 18 | 88.9 | 7 to 18 |
| Radius-Distal | 3 | 19 | 22 | 86.4 | 7 to 18 |
| Tibia-Proximal | 4 | 14 | 18 | 77.8 | 7 to 18 |
| Ulna-Distal | 0 | 0 | 0 | 0.0 | 7 to 18 |
| TOTAL Stage 4/5 | 79 | 229 | 308 | 74.4 | 7 to 18 |

Age stages follow Munro et al. (2009).

**Table B.** *Gazella* tooth wear and eruption data from MPPNB Yiftah’el.

| Age Stage | Age | MNE |
| --- | --- | --- |
| Stage I | 0-3 mos | 0 |
| Stage II | 3-7 mos | 0 |
| Stage III | 7-18 mos | 7 |
| Stage IV | 18-36 mos | 5 |
| Stave V+VI | 36-96 mos | 5 |
| Stage VII | 96+ | 0 |

Age stages follow Munro et al. (2009).

**Table C.** *Capra* fusion data for MPPNB Yiftah’el.

|  | MNE | MNE | MNE |  | Fusion Age (mos) |
| --- | --- | --- | --- | --- | --- |
| Bone Portion | Unfused | Fused | Total | %Fused | Zeder 2006 |
| Radius-Proximal | 0 | 16 | 16 | 100.0 | 0 to 6 |
| TOTAL STAGE 1 | 0 | 16 | 16 | 100.0 | 0 to 6 |
| 1st Phalanx-Prox | 4 | 47 | 51 | 92.2 | 6 to 12 |
| 2nd Phalanx-Prox | 2 | 26 | 28 | 92.9 | 6 to 12 |
| Humerus-Distal | 1 | 27 | 28 | 96.4 | 6 to 12 |
| Pelvis-Acetabulum | 1 | 12 | 13 | 92.3 | 6 to 12 |
| Scapula-Glenoid | 3 | 18 | 21 | 85.7 | 6 to 12 |
| TOTAL STAGE 2 | 11 | 130 | 141 | 92.2 | 6 to 12 |
| 1st Phalanx-Prox | 4 | 47 | 51 | 92.2 | 12 to 18 |
| 2nd Phalanx-Prox | 2 | 26 | 28 | 92.9 | 12 to 18 |
| TOTAL STAGE 3 | 6 | 73 | 79 | 92.4 | 12 to 18 |
| Tibia-Distal | 2 | 6 | 8 | 75.0 | 18 to 30 |
| Metapodial-Distal | 8 | 12 | 20 | 60.0 | 18 to 30 |
| TOTAL STAGE 4 | 10 | 18 | 28 | 64.3 | 18 to 30 |
| Tibia-Proximal | 2 | 3 | 5 | 60.0 | 30 to 48 |
| Femur-Proximal | 1 | 3 | 4 | 75.0 | 30 to 48 |
| Femur-Distal | 3 | 6 | 9 | 66.7 | 30 to 48 |
| Calcaneum | 6 | 5 | 11 | 45.5 | 30 to 48 |
| Ulna-Proximal | 4 | 9 | 13 | 69.2 | 30 to 48 |
| Radius-Distal | 3 | 5 | 8 | 62.5 | 30 to 48 |
| TOTAL STAGE 5 | 19 | 31 | 50 | 62.0 | 30 to 48 |
| Humerus-Proximal | 2 | 1 | 3 | 33.3 | 7 to 18 |
| TOTAL STAGE 6 | 2 | 1 | 3 | 33.3 | 7 to 18 |

**Table D.** *Capra* tooth wear and eruption data from MPPNB Yiftah’el.

| Age | Payne Stage | MNE |
| --- | --- | --- |
| 0-2 mos | A | 0 |
| 2-6 mos | B | 0 |
| 6-12 mos | C | 1 |
| 1-2 yrs | D | 3 |
| 2-3 yrs | E | 2 |
| 3-4 yrs | F | 3 |
| 4-8 yrs | G-H | 4 |
| 8-10 yrs | I | 0 |

Age stages follow Payne (1973).

**Table E**. *Sus* fusion data for MPPNB Yiftah’el.

|  | MNE | MNE | MNE |  | Fusion Age (mos) |
| --- | --- | --- | --- | --- | --- |
| Bone Portion | Unfused | Fused | Total | %Fused | Silver 1967 |
| Pelvis-Acetabulum | 2 | 1 | 3 | 33.33 | 0 to 12 |
| Scapula-Glenoid | 1 | 6 | 7 | 85.71 | 0 to 12 |
| Humerus-Distal | 4 | 7 | 11 | 63.64 | 0 to 12 |
| Radius-Proximal | 0 | 4 | 4 | 100.00 | 0 to 12 |
| 2nd Phalanx-Prox | 2 | 16 | 18 | 88.89 | 0 to 12 |
| TOTAL | 9 | 34 | 43 | 79.07 | 0 to 12 |
| Metacarpal-Distal | 4 | 6 | 10 | 60.00 | 12 to 24 |
| 1st Phalanx-Prox | 6 | 15 | 21 | 71.43 | 12 to 24 |
| Tibia-Distal | 2 | 4 | 6 | 66.67 | 12 to 24 |
| TOTAL | 12 | 25 | 37 | 67.57 | 12 to 24 |
| Metatarsal-Distal | 1 | 1 | 2 | 50.00 | 24 to 36 |
| Fibula-Distal | 0 | 1 | 1 | 100.00 | 24 to 36 |
| Calcaneum | 9 | 6 | 15 | 40.00 | 24 to 36 |
| TOTAL | 10 | 8 | 18 | 44.44 | 24 to 36 |
| Tibia-Proximal | 2 | 1 | 3 | 33.33 | 36 to 48 |
| Humerus-Proximal | 2 | 0 | 2 | 0.00 | 36 to 48 |
| Radius-Distal | 2 | 0 | 2 | 0.00 | 36 to 48 |
| Ulna-Proximal | 6 | 1 | 7 | 14.29 | 36 to 48 |
| Femur-Proximal | 5 | 4 | 9 | 44.44 | 36 to 48 |
| Femur-Distal | 6 | 0 | 6 | 0.00 | 36 to 48 |
| Fibula-Prox | 0 | 0 | 0 | ND | 36 to 48 |
| TOTAL | 23 | 6 | 29 | 20.69 | 36 to 48 |

**Table F.** *Bos* fusion data for MPPNB Yiftah’el.

|  | MNE | MNE | MNE |  | Fusion Age (mos) |
| --- | --- | --- | --- | --- | --- |
| Bone Portion | Unfused | Fused | Total | %Fused | Silver 1967 |
| Pelvis-Acetabulum | 1 | 3 | 4 | 75.00 | 0 to 24 |
| Scapula-Glenoid | 1 | 1 | 2 | 50.00 | 0 to 24 |
| Humerus-Distal | 1 | 3 | 4 | 75.00 | 0 to 24 |
| Radius-Proximal | 0 | 3 | 3 | 100.00 | 0 to 24 |
| 1st Phalanx-Prox | 2 | 14 | 16 | 87.50 | 0 to 24 |
| 2nd Phalanx-Prox | 12 | 22 | 34 | 64.71 | 0 to 24 |
| TOTAL | 17 | 46 | 63 | 73.02 | 0 to 24 |
| Tibia-Distal | 1 | 1 | 2 | 50.00 | 24 to 48 |
| Metapodial-Distal | 2 | 11 | 13 | 84.62 | 24 to 48 |
| Humerus-Proximal | 0 | 0 | 0 | ND | 24 to 48 |
| Ulna-Proximal | 0 | 0 | 0 | ND | 24 to 48 |
| Femur-Proximal | 1 | 1 | 2 | 50.00 | 24 to 48 |
| Radius-Distal | 0 | 0 | 0 | ND | 24 to 48 |
| Femur-Distal | 1 | 1 | 2 | 50.00 | 24 to 48 |
| Tibia-Proximal | 1 | 1 | 2 | 50.00 | 24 to 48 |
| Calcaneum | 5 | 1 | 6 | 16.67 | 24 to 48 |
| TOTAL | 11 | 16 | 27 | 59.26 | 24 to 48 |
