# Supplementary material for: Human Hunting and Nascent Animal Management at Middle Pre-Pottery Neolithic Yiftah'el, Israel
Source: PLoS One. 2016 Jul 6;11(7):e0156964. doi: 10.1371/journal.pone.0156964 (PMC4934702; doi:10.1371/journal.pone.0156964)
Supplement: S2 Table — (DOCX) [file pone.0156964.s003.docx]

**S2 Table. Raw measurements for *Gazella*, *Capra*, *Sus* and *Bos* from MPPNB Yiftah'el. Measurement abbreviations follow von den Driesch (1976).**

**Table A. Raw measurements for *Gazella* from MPPNB Yiftah'el**

| ELEMENT | MEASUREMENT | |  |  |  |  |  |  |
| --- | --- | --- | --- | --- | --- | --- | --- | --- |
| SCAPULA | BG | 17.36 | 17.45 | 18.15 | 18.73 | 18.8 | 18.86 | 19.02 |
|  |  | 19.16 | 19.43 | 19.71 | 19.75 | 19.81 | 19.85 | 20.02 |
|  |  | 20.11 | 20.18 | 20.19 | 20.23 | 20.37 | 20.54 | 20.61 |
|  |  | 20.62 | 20.83 | 20.84 | 20.89 | 21.44 | 21.6 | 21.72 |
|  |  | 21.76 | 22.18 | 22.37 | 22.42 | 22.57 | 22.58 | 22.74 |
|  |  | 22.81 | 23.25 | 23.6 |  |  |  |  |
| HUMERUS | Bd | 23.14 | 23.55 | 24.13 | 24.64 | 24.84 | 25.06 | 25.13 |
|  |  | 25.15 | 25.18 | 25.55 | 25.57 | 25.69 | 25.89 | 26.02 |
|  |  | 26.02 | 26.18 | 26.24 | 26.44 | 26.44 | 26.5 | 26.76 |
|  |  | 26.79 | 26.81 | 26.9 | 26.96 | 26.97 | 27.06 | 27.09 |
|  |  | 27.14 | 27.16 | 27.24 | 27.31 | 27.43 | 27.44 | 27.58 |
|  |  | 27.66 | 27.66 | 27.68 | 27.71 | 27.95 | 28.01 | 28.16 |
|  |  | 28.16 | 28.19 | 29.3 | 29.68 | 29.78 | 30.66 |  |

**Table B. Raw measurements for *Capra* from MPPNB Yiftah'el**

| ELEMENT | MEASUREMENT |  |  |  |  |  |  |  |
| --- | --- | --- | --- | --- | --- | --- | --- | --- |
| SCAPULA | BG | 26.44 | 25.97 | 21.14 | 24.05 | 21.78 | 21.9 |  |
|  |  | 27.07 | 19.77 | 24.46 |  |  |  |  |
| HUMERUS | Bd | 31.63 | 34.71 | 31.85 | 35.13 | 35.11 | 34.01 |  |
|  |  | 39.67 | 29.55 | 32.62 | 40.89 | 29.23 | 32.49 |  |
|  |  | 29.21 | 31.57 |  |  |  |  |  |
| RADIUS | Bp | 33.88 | 34.89 | 35.75 | 27.73 | 30.38 | 42.75 |  |
|  |  | 40.15 | 29.87 | 29.24 | 37.33 |  |  |  |
| RADIUS | Bd | 27.89* | 30.42 | 34.48 | 33.38 | 28.2 | 29.73 |  |
|  |  | 33.47 |  |  |  |  |  |  |
| ULNA | BPC | 21.48 | 26.25 | 26.58 | 20.61 |  | 21.57 | 30.04 |
| METACARPAL | Bp | 21.02 | 23.02 | 23.88 | 24.15 | 26.61 | 26.65 | 27.99 |
| METACARPAL | Bd | 30.83 | 28.58 | 29.82 |  |  |  |  |
| PHALANX 1 | Bp | 13.99 | 14.4 | 16.41 | 15.12 | 14.42 | 13.92 | 14.62 |
|  |  | 15.61 | 16.35 | 16.79 | 11.84 | 14.25 | 15.32 | 15.48 |
| FEMUR | Bp | 44.5 | 45.2 | 55.02 | 44.99 |  |  |  |
| FEMUR | Bd | 36.65 | 47.72 | 35.28 | 41.08* |  |  |  |
| TIBIA | Bp | 37.19 |  |  |  |  |  |  |
| TIBIA | Bd | 28.92 | 33.54 | 27.43 |  |  |  |  |
| CALCANEUS | GB | 19.85 | 18.92 | 18.71 |  |  |  |  |
| METATARSUS | Bp | 22.02 | 21.48 | 22.06 | 20.29 | 21.22 | 20.12 |  |
| METATARSUS | Bd | 32.04 | 26.24 | 27.95 |  |  |  |  |

* Unfused bone

**Table C. Raw measurements for *Sus* from MPPNB Yiftah'el**

| ELEMENT | MEASUREMENT |  |  |  |  |  |  |
| --- | --- | --- | --- | --- | --- | --- | --- |
| TIBIA | Bd | 34.2 | 31.97 |  |  |  |  |
| HUMERUS | Bd | 55.74 | 42.67 | 46.06 | 45.98 |  |  |
| SCAPULA | BG | 27.19 | 24.84 | 23.54 | 23.07 | 27.61 | 29.67 |
| ULNA | BPC | 22.88 | 24.48 | 23.6 | 22.43 |  |  |
| RADIUS | Bp | 35.42 | 31.73 | 31.03 |  |  |  |

**Table D. Raw measurements of *Bos* from MPPNB Yiftah'el**

| ELEMENT | MEASUREMENT |  |  |  |  |  |  |  |
| --- | --- | --- | --- | --- | --- | --- | --- | --- |
| PHALANX 2 | Bp | 28.4 | 32.3 | 33.12 | 33.41 | 33.52 | 33.69 | 34.18 |
|  |  | 34.28 | 35.01 | 35.15 | 35.84 | 37.89 | 38.34 | 38.88 |
|  |  | 41.62 |  |  |  |  |  |  |
| PHALANX 1 | Bp | 27.54 | 32.15 | 32.81 | 34.09 | 34.15 | 35.71 | 35.74 |
|  |  | 36.18 | 36.98 | 38.6 |  |  |  |  |
| HUMERUS | Bd | 92.01 | 85.47 |  |  |  |  |  |
| RADIUS | Bp | 86.2 |  |  |  |  |  |  |
| METACARPUS | Bd | 64.03 | 70.16 | 77.07 |  |  |  |  |
| METACARPUS | Bp | 62.9 |  |  |  |  |  |  |
| METATARSUS | Bd | 70.06 |  |  |  |  |  |  |
| METATARSUS | Bp | 53.04 | 59.65 | 59.55 |  |  |  |  |
